# Objective quantification and spatial mapping of cataract with a Shack-Hartmann wavefront sensor

**DOI:** 10.1038/s41598-020-69321-3

**Published:** 2020-07-28

**Authors:** Csaba Tamás Holló, Kata Miháltz, Máté Kurucz, Anita Csorba, Kinga Kránitz, Illés Kovács, Zoltán Zsolt Nagy, Gábor Erdei

**Affiliations:** 1 Department of Atomic Physics, Budapest University of Technology and Economics, Budapest, Hungary; 2 Department of Ophthalmology, Hietzing Hospital with Neurological Center Rosenhügel, Vienna, Austria; 3 Department of Ophthalmology, Semmelweis University, Budapest, Hungary; 4 Department of Ophthalmology, Weill Cornell Medical College, New York, NY USA

**Keywords:** Medical research, Optical physics, Imaging techniques, Characterization and analytical techniques, Imaging and sensing, Lens diseases

## Abstract

Based on wavefront sensor images an objective and quantitative method is presented for characterising cataract. By separating direct and scattered light in the focal plane of the microlenses, the new procedure is able to make two-dimensional maps of the spatial variation of scattering properties in the crystalline lens, and also provides a single figure descriptive for the whole eye. The developed evaluation algorithm successfully quantifies cataract, especially that of nuclear type. To demonstrate its operation, a custom-built measurement setup was constructed using a Shack-Hartmann wavefront sensor with $40 \times 32$ microlenses to capture 12-bit images of the pupil plane, and a superluminescent diode of 830 nm wavelength as a light source. Slit-lamp clinical measurements served as reference for calibration and to estimate the accuracy of the new method. The tests were carried out on 78 eyes with cataract in different progression state ranging from healthy to above 5 on the LOCS III scale. The residual error of the calibration (i.e. the standard deviation of difference between clinical reference and our algorithmic characterisation) turned out to be $\pm 0.29$ category on the LOCS III N scale, which approximates the $\pm 0.33$ precision of classic cataract measurements carried out with the greatest care.

## Introduction

Formerly an eye disease of elderly people, cataract surgery is nowadays very often indicated for those being in the middle age too. This happens partly because of the increasing demand for good visual quality, a significant improvement in surgical technique, as well as a growing exposure to cataractogenic risk factors. These are various: diabetes, elevated levels of ultraviolet/infrared radiation, prior intraocular surgery, oxidative stress, just to mention a few. Resulting in the partial or total opacity of the crystalline lens, cataract can eventually cause significant visual impairment, and thus, decreased life quality. Although antioxidants have been prompted as therapeutic options to delay or prevent cataract progression, symptomatic cataract is a surgical disease: the standard of care in cataract surgery is a small-incision phacoemulsification with foldable intraocular lens implantation. The negative impact of a cataract on visual quality is often reported by the patients as blurry vision or glare. As mankind gradually adapts to the processing of an ever-increasing amount of visual information, nowadays people tend to notice even a delicate decline in visual quality, the cause of which is not always straightforward to identify. Although several types of tests exist to assess the visual function, currently there is no means to separate the effect of cataract on a patient’s visual status or functional ability from other disorders. For instance, the evaluation of visual impairment as a result of cataract is especially challenging in cases when lens opacity coexists with some macular disease (such as age-related macular degeneration). There are other fields too, that could benefit from a sensitive, objective and quantitative cataract measuring method: e.g. the research of cataract-reversing eye drops^1^ and intraocular lens design, which can gain useful information from the accurate follow-up of secondary cataract that often occurs after implantation^2, 3^.

Opacity impairs vision by intensifying forward light scattering, thereby decreasing image contrast at the retina. Correspondingly, the traditional diagnosis of cataract is based on measuring the scattering properties of the crystalline lens. The most common clinical test is the slit-lamp measurement^4^, in which cataract is quantified by illuminating the eye obliquely with a narrow line-shaped light-source, while the ophthalmologist inspects the crystalline lens from the front through a microscope. Visual observation is then compared to a standard reference image sequence to determine the grade of progression; e.g. in the case of nuclear cataract there are six categories (plus the scatter-free case). In addition to this coarse figure, the method is also strongly subjective, since the diagnosis is affected by the personal judgement and specific experience of the examiner. Further drawbacks are that by using a slit-lamp only backward scattering can be examined instead of the physically relevant effect, and the provided metric is not directly related to the visual loss of the patient^5^. The latter problems are eliminated in case of the C-Quant^6^ equipment, which investigates how a disturbing source affects the visibility of a target viewed by the patient. Although it provides a better way of quantification, subjectivity (in this case that of the subject) is still an issue. Fully objective and quantitative testing solutions also exist. A double-pass setup was commercialized to examine the image of a retinal point source distorted by ocular scattering^7, 8^. The method needs to be carefully corrected for refractive errors and only measures overall cataract without providing a spatial map of the pupil. Scheimpflug-camera^9, 10^ is also a common tool in ophthalmology with a long history of cataract detection, however the method shares the disadvantage of slit-lamp measurements that examine backward scattered light. Optical Coherence Tomography^11–13^ (OCT) was also suggested as a potential tool for cataract examination. A comparison of all these methods can be found in Kamiya et. al.’s paper^14^.

An interesting approach found in the literature^15^ quantifies cataract by using a Shack-Hartmann wavefront sensor^16^. Diagnostic apparatuses applying such a device have been developed and are widely available for the measurement of monochromatic wavefront aberration of the eye. In their study, Donelly at al. attempted to correlate visual acuity with the scattering properties of the eye by using a custom equipment, but no reassuring correspondence was found. They pointed out that their optical system was unable to detect all scattered light, concluding that this may have been one of the reasons of the negative result. The method was also tested on clinical (Zeiss-made WASCA) aberrometers^17^ as well, using C-Quant as a reference. The study could reveal some correlation between the two measurements, though the lack of exposure control possibility strongly reduced the interpretability of the captured images.

Our aim was to develop an objective and quantitative method to characterize cataract by measuring forward scattering in the eye with a Shack-Hartmann wavefront sensor. In order to have complete control over design parameters as well as operational settings, we constructed a custom measurement setup. For the evaluation of raw wavefront sensor images we developed a novel algorithm that quantifies cataract by decimal values interpreted on the LOCS III N scale. Structure of the apparatus and details of the image-processing steps are described. Calibration of our method by clinical measurements are presented.

## Methods

### Working principle

**Figure 1:**
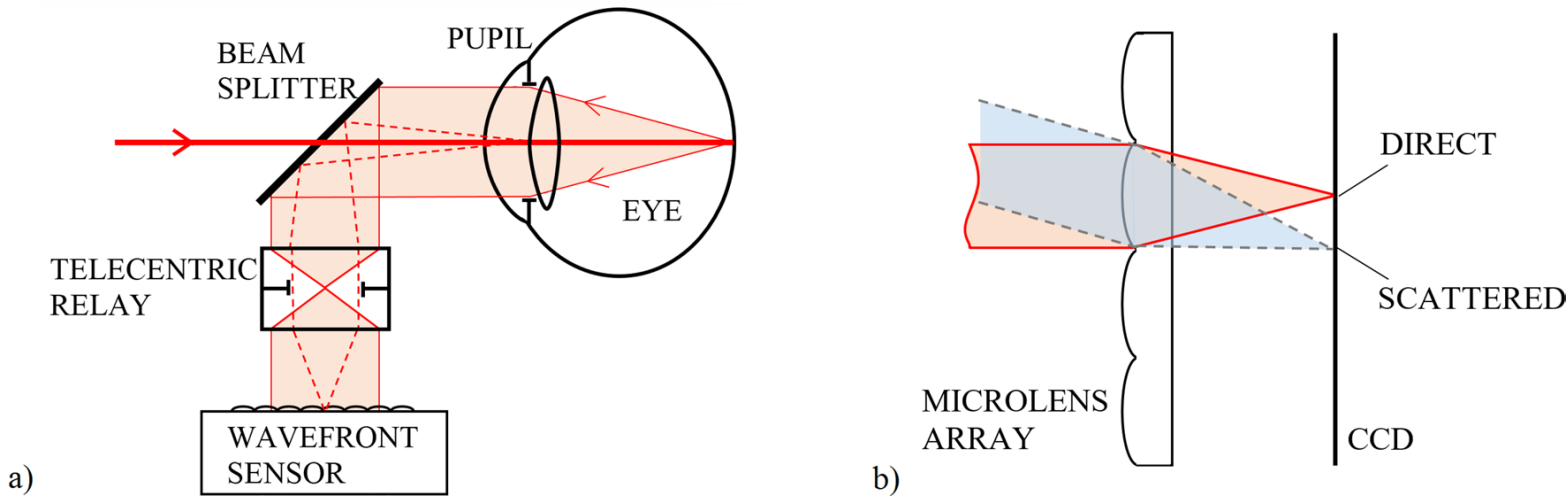
Working principle: **(a)** Single-pass analysis of the eye. The pupil plane together with scattered light are projected onto the microlens array of a wavefront sensor. **(b)** Direct (non-scattered) and scattered light is focused at different locations on the CCD detector array forming the direction characteristics of scattered light incident locally on the actual microlens.

A scheme of the single-pass setup used in our measurement is depicted in Fig. 1a. A narrow collimated infrared beam illuminates the eye that focuses it at the retina. Here the light is reflected back from the aqueous humor/inner limiting membrane (ILM) interface and gets slightly scattered by the surface roughness of the latter. The resulting light spot forms a point source at the back of the eye that illuminates the entire pupil internally. As the back-scattered beam travels through the crystalline lens again, it suffers volume scattering due to cataract, containing information about the forward scattering of the eye. (We call the portion left unscattered by cataract as “direct light”.) Then, a telecentric relay objective projects the beam onto the wavefront sensor’s microlens array, the lenslets of which having focused at the detector surface. The relay has a double-role. First, it images the pupil of the eye to the plane of the microlens array, providing a spatial mapping. Second, due to its telecentricity, the objective keeps the plane-wave components of light scattered by cataract collimated when reaching the wavefront sensor. Consequently, in the focal plane of each microlens the local direction characteristics (i.e. Fourier-transform) of scattered light is formed, see Fig. 1b. In other words, the irradiance (*E*) measured at any point in the focal plane of a microlens is proportional to the radiant intensity (*I*) measured in the corresponding direction at the eye.

The image of the illuminating beam reflected by the ILM (i.e. the direct light) presents itself as a small bright spot in the focal plane of a microlens, whereas light scattered by cataract forms a larger, weaker distribution around it. (Practically, the focal spot of direct light is an image of the light spot at the retina.) As light scattering increases in the crystalline lens the ratio of peak irradiance of the focal spot and the surrounding distribution changes, thus cataract can be detected. Unfortunately, light is scattered not only by the surface of the ILM, but also by the complex internal structure of the retina (and from other places in the human eye far from the pupil plane). The result is a massive volumetric scattering that constitutes a constant background around the focal spots of the microlenses even in case of a healthy eye. This causes a kind of offset, that should be subtracted from the measured irradiance values. Since each microlens of the wavefront sensor observes a separate portion of the pupil, a spatial map of cataractous scattering can be determined in the eye.

### Equipment and software

**Figure 2:**
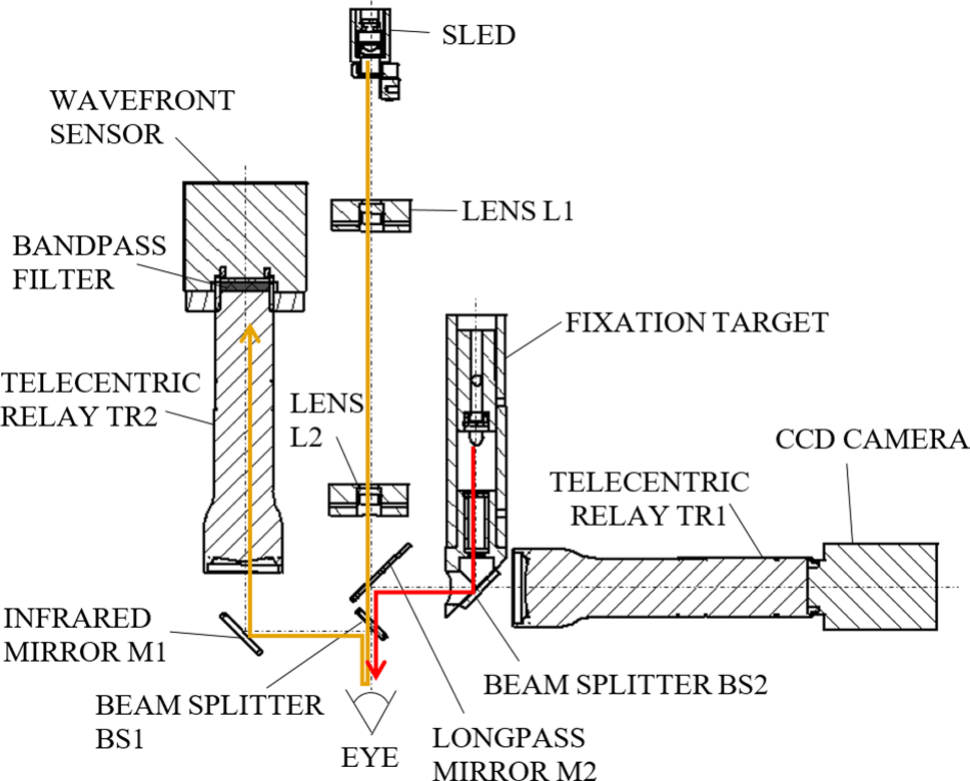
Scheme of the experimental setup. Orange path: infrared, used for scattering measurements. Red path: visible red, used to project a holographic fixation target into the eye for its stabilisation.

The experimental setup of our measurement is shown in Fig. 2. The collimated continuous infrared illumination of 830 nm central wavelength is produced by a Hamamatsu L8414-41 superluminescent LED (SLED). The beam diameter at the pupil is 0.6 mm, the radiant power is 12 $\upmu$W. These parameters allow the classification of the measurement device as a Group 1 equipment according to the ISO 15004-2:2007 standard, meaning that its medical application does not pose any light hazard. The TR2 telecentric relay (Edmund Optics 0.5$\times$ SilverTL) maps the pupil to the microlens plane in the Imagine Optic HASO3-32 wavefront sensor, which is protected from unwanted light by an interference filter of 40 nm bandwidth centered at 850 nm. The microlens array contains $40 \times 32$ microlenses with a pitch of 153 $\upmu$m and focal length of 5.0 mm. The CCD (charge-coupled device) detector array of the wavefront sensor has $656 \times 494$ pixels of 9.91 $\upmu$m size, thus an area of $15.4 \times 15.4$ pixels belongs to one microlens in average. This corresponds to a total scattering angle of ±7.6 mrad at the eye, where the angular size of one CCD pixel is 1.0 mrad. Telecentric relay TR1 (the same type as TR2) images the pupil plane to an AVT Marlin F201B CCD camera, which is used for pupil monitoring and to adjust the position of the equipment relative to the patient’s eye. Finally, the fixation target projects a red holographic cross into the eye, assisting the patient to reduce eye movement by staring at it during the measurement. The focal position of the cross is adjustable in the ±5 D range, so that patients can see it sharply without prescription glasses.

The measurement is computer-controlled via a MatLAB macro. The software is capable of grabbing/displaying raw images simultaneously taken by the pupil camera and the wavefront sensor. It also controls the SLED illumination and automatically turns it on/off as necessary. The number of images captured in a sequence is adjustable.

### Separation of scattered and direct light in the captured images

Light scattering in the eye is sufficiently small even in case of cataract grade 5 to result in negligible crosstalk between the images of two neighboring microlenses (for an explanation see the next subsection). Consequently, the light distribution represented by the raw wavefront sensor image can be evaluated for each individual microlens separately. According to our theory described above, the irradiance distribution of a microlens spot consist of three parts: 1) the direct beam, which is not scattered at all, 2) light scattered in the crystalline lens, and 3) a constant background originating mainly from volume scattering in the retina.

The basis of separating parts 1), 2), 3) from each other is that the width of the focal spot of direct light is practically independent of the examined eye, but it is rather determined by the optical properties of the microlenses (geometric aberrations, surface roughness, light diffraction on their apertures etc.) In order to support this claim we investigated the potential effect of ocular refractive errors on the shape of the spots. The most common and dominating aberration in the human eye is defocus, thus we used it for our demonstration. We artificially illuminated into the measuring equipment from the “eye side” with a collimated SLED identical to its internal light source, which models the examination of a scatter-free ideal eye. By adjusting the collimator lens-SLED distance we could modify the curvature of the output spherical wavefront, simulating defocus. The spot sizes measured by the wavefront sensor were characterised by their FWHM (full-width-at-half-maximum) value. In a defocus range of [−5 D, 5 D] the average FWHM (computed for the whole wavefront sensor) systematically increased by 0.24 mrad, whereas the standard deviation was 0.18 mrad within one image. Since both variations are one order of magnitude smaller than the focal spot sizes in case of a typical eye (i.e. FWHM 3.4 mrad), we can neglect their effect.

In order to take realistic scattering into account too, we also repeated the above analysis in vivo with a healthy eye. In this case defocus was introduced by adjusting the fixation target in a range of [−5 D, 0 D]. The subject was asked to keep its image sharp on his retina by crystalline lens accommodation, which corresponds to the case as if he normally wears glasses, but now looks into the device without it. The average FWHM changed in a 0.15 mrad range without any correlation with the defocus value, and the standard deviation of FWHM was 0.32 mrad within one image. These are very close to the formerly determined values, supporting our claim that in case of a given measuring equipment the direct light reflected by the retina is focused by the microlenses into spots, the size of which can be regarded as independent of the examined eye’s aberrations.

### Quantification of local scattering

The initial step of our algorithm is to localize the center of each focal spot of direct light. First, every pixel is labelled if its irradiance exceeds the mean value in its neighborhood of 8 pixel radius (i.e. the characteristic size of detector surface behind a microlens) by 20% of the peak-to-valley value measured in this environment. Then, the centroid of the irradiance distribution of the labelled pixels is determined to represent the center of the focus spot behind a given microlens. Saturated pixels and their 10 pixel-wide neighborhood are excluded from the evaluation.

In case of each microlens we determine the 1), 2) and 3) constituent parts of the focal spot irradiance distributions by curve fitting using an empirical function formulated as (1). In this equation $A_1$ is the amplitude of the direct beam, $A_2$ is that of light scattered by the crystalline lens, and $z_0$ represents the constant background. In (1) $\varphi$ denotes the polar angle relative to the optical axis of the eye, $\sigma _1$ describes the $1/e^2$ irradiance radius of the directly reflected beam and $\sigma _2$ the same for scattered light.

1 $$\begin{aligned} E(\varphi )=A_1\cdot \exp \left( {-2\left( {\frac{\varphi }{\sigma _1}}\right) ^2}\right) +A_2\cdot \exp \left( {-2\left( {\frac{\varphi }{\sigma _2}}\right) ^2}\right) +z_0 \end{aligned}$$

**Figure 3:**
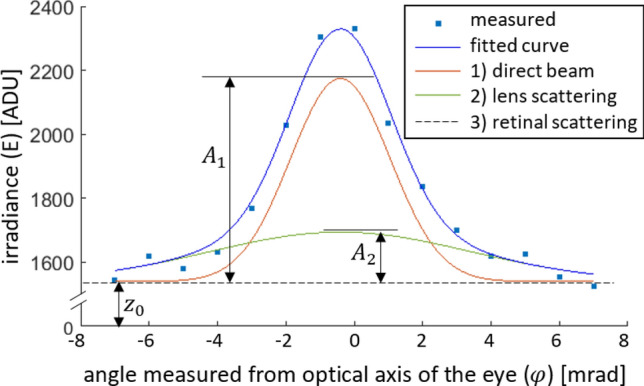
Measured radial irradiance distribution of a focus spot together with its fitted regression curve. Decomposition of light into direct and scattered parts are also indicated. ADU means analog-to-digital unit with a maximum value of 4095.

Our measurements showed that only $A_1$ and $A_2$ change as scattering grows in the eye, but $\sigma _1$ and $\sigma _2$ (i.e. the widths of the bell-shaped curves in Fig. 3) remain the same. Their values were determined in the following way. Allowing every parameter to change in Eq. (1), first we performed curve fitting on the wavefront sensor data of several healthy eyes, in which crystalline lens scattering was much less than in cataractous eyes (i.e. the direct beam dominated), and fixed $\sigma _1$ at its most frequent value (mode): 2.89 mrad. (The same result can also be obtained by using the scatter-free artificial eye model presented in the previous subsection.) Then, focal spots of eyes with severe cataract were fitted, with $\sigma _1$ kept constant, to determine the most frequent value of $\sigma _2$, which was 7.55 mrad. This means that approximately 91% of light falling on a Shack-Hartmann microlens is captured by the square-shaped sensor area behind it. Only the rest, i.e.  9% is distributed over adjacent sensor areas, which indicates a low crosstalk between neighboring lenslets. We made all subsequent calculations with these two particular $\sigma$ values. An example for the irradiance distribution of a focal spot is shown in Fig. 3. We consider all 1), 2), 3) distributions to be axially symmetric, the average FWHM value of the direct light’s focal spots (see red curve) is 3.4 mrad. The average value of the root-mean-square fitting error *ERR*, defined by Eq. (2) is 3.5%. In Eq. (2) the summing index *i* runs through the pixels behind one microlens, $E(\varphi _i)$ and $E_i$ are the fitted and measured values, respectively.

2 $$\begin{aligned} ERR= \frac{1}{A_1+A_2+z_0}\sqrt{\frac{1}{n}\sum _{i=1}^{n}\left( E\left( \varphi _i\right) -E_i\right) ^2} \end{aligned}$$

In order to describe the magnitude of scattering locally at a given microlens, we introduced a so-called scattering parameter (*S*). According to its definition (Eq. 3), the value of *S* grows with increasing scattering. Spatial mapping of *S* is done by evaluating all focal spots of the microlens array for a given eye. Due to the application of a relay lens, the spatial resolution at the eye becomes the size of one microlens divided by the magnification of TR2 (i.e. 0.5), resulting in 0.31 mm in our setup.

3 $$\begin{aligned} S\equiv A_2/A_1 \end{aligned}$$

### Global quantity to characterize the cataract of a given eye

**Figure 4:**
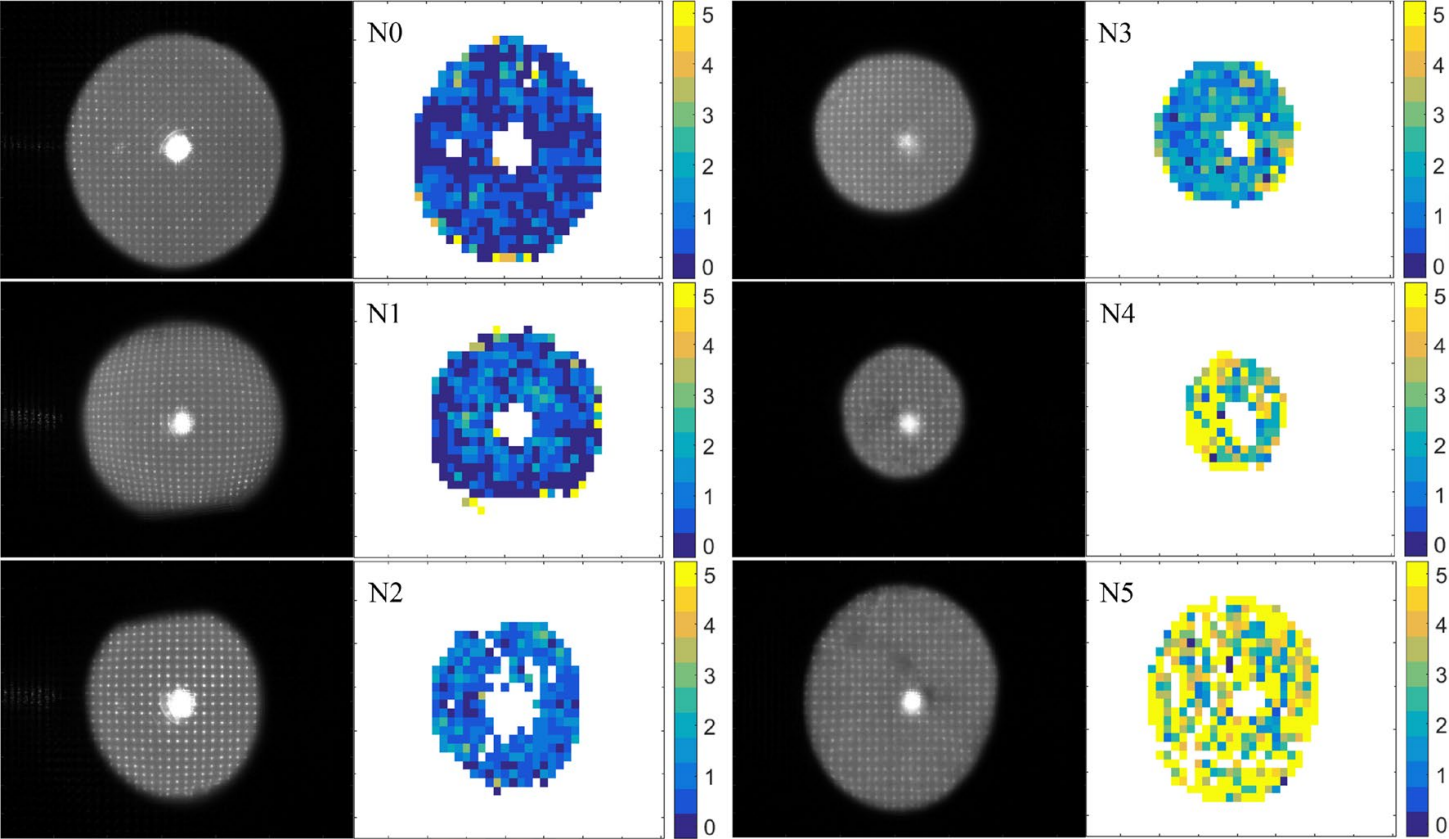
Exemplary wavefront sensor images and the corresponding pupil maps of the scattering parameter (*S*) at separate stages of nuclear (N-type) cataract. Coloring indicates the most common values of *S* in terms of LOCS III categories.

Though scattering maps of the pupil are very informative (see Fig. 4 in the “Results” section), the traditional clinical practice needs a single quantity that characterizes cataract of a specific eye as a whole. It is not a straightforward task to fulfill this requirement, since cataract can occur either in some local region of the crystalline lens, or uniformly in its entire volume, which gives rise to a diversity of *S* values even in a single eye. The distribution of these (i.e. the frequency density vs. *S* histogram) thus changes from eye to eye. Examples of such histograms can be seen in the (a) and (c) subfigures of Fig. 6, revealing their skewed, Gaussian-like nature. Our aim was to find a statistical measure of these distributions that changes monotonously as scattering increases (linearity was preferred). For this purpose we created the following special regression function, with which *S*-histograms can be adequately fitted using a very small number of independent parameters:

4 $$\begin{aligned} f(S)=\frac{c}{\sigma }\exp \left[ {-2\left( \frac{S-S_0}{\sigma }\cdot \left( \frac{1}{1+\exp {\frac{k(S-S_0)}{\sigma }}}+\frac{1}{2}\right) \right) ^2}\right] . \end{aligned}$$

In Eq. (4) $S_0$ denotes the peak position, i.e. the mode of *S*, $\sigma$ describes the width of the distribution, *k* accounts for its skewness, and *c* is a *k*-dependent normalization constant that ensures the integral to be unity. As we fitted this function to many histograms obtained from different eyes, it turned out that skewness hardly changes, thus *k* can be kept constant at a value of 1.64. From this we calculated numerically the value of the normalization constant: $c=0.6609$. In this way the number of free parameters necessary to describe scattering in a given eye reduced to two: $S_0$ and $\sigma$. We found that eyes with different degrees of cataract can be better separated in terms of $S_0$ than $\sigma$, see e.g. N0 and N1 groups in Fig. 6d, therefore we chose $S_0$ as the quantity to characterize the cataract of the whole eye.

### Subject pool

The clinical experiments were performed at the Department of Ophthalmology, Semmelweis University, Budapest, Hungary. The subjects were free of any known eye disease except cataract and low-order refractive errors (power and astigmatism), any other retinal and corneal abnormalities were excluded. All subjects underwent pupil dilation before the test. The measurements were carried out with the co-operation of a qualified ophthalmic clinical officer, who determined the LOCS III classification of each eye by using a slit-lamp according to the standard clinical protocol. This LOCS III categorization served as our reference during the subsequent evaluation of wavefront sensor images. We followed the tenets of the seventh revision of the Declaration of Helsinki (2013) in our study. All subjects gave their written informed consent. Experiments were officially approved by the National Institute of Pharmacy and Nutrition which is the competent authority in Hungary, permission number: OGYÉI/39200/2018, date of registration 4th September 2018.

By the selection of subjects we strove for covering the whole LOCS III scale, especially focusing on the case of nuclear cataract, since it is the most common type. The group of patients with cataractous eyes consisted of 45 persons, their age ranging from 51 to 89 (average: 69). In total 59 eyes in this group could be measured, from this 52 eyes were of N-type. 13 healthy young participants with an average age of 24 in a range of 23 to 26 was used as a control group. None of them had diagnosable cataract, i.e. all the 26 measured eyes in the control group were categorised as N0, according to the LOCS III scale.

## Results

### Measurements

Examples of raw wavefront sensor images are shown in Fig. 4: it can be seen as the focal spots are getting more blurred in case of eyes with more pronounced cataract. Beside each image the corresponding pupil map of *S* parameters quantifies the level of scattering at separate regions of the pupil. The central bright spot comes from the light source as it reflects off the cornea.

**Figure 5:**
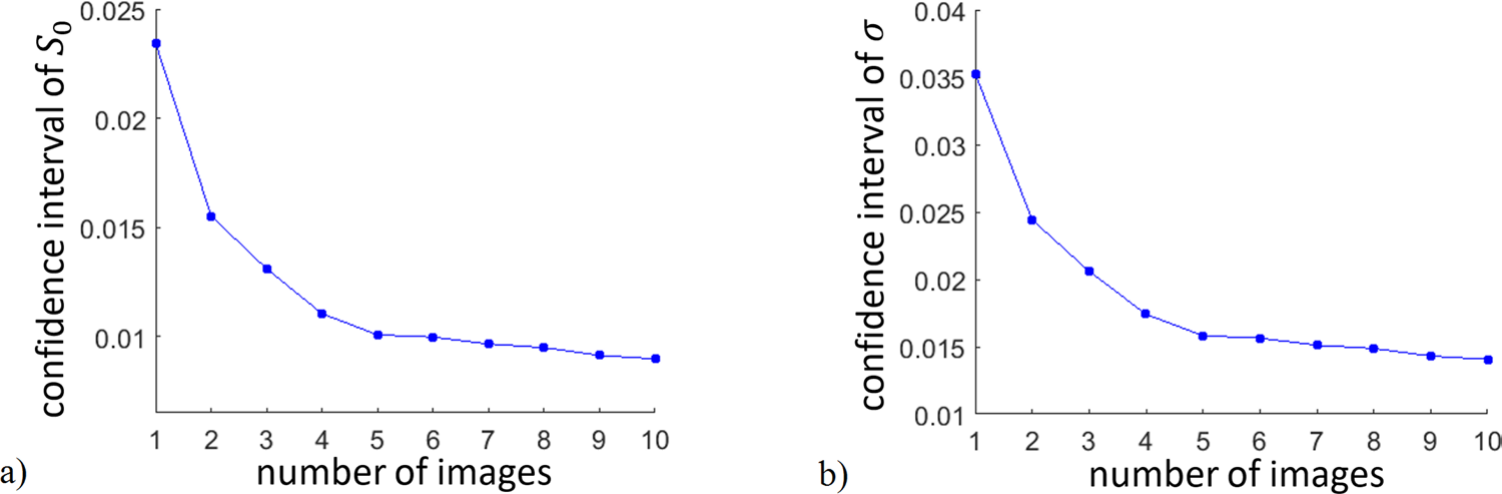
95% confidence interval radius of fitting parameters in Eq. (4): **(a)** $S_0$ and **(b)** $\sigma$, calculated as averages using different number of images acquired from the same eye. Individual results have been averaged for all measured eyes.

**Figure 6:**
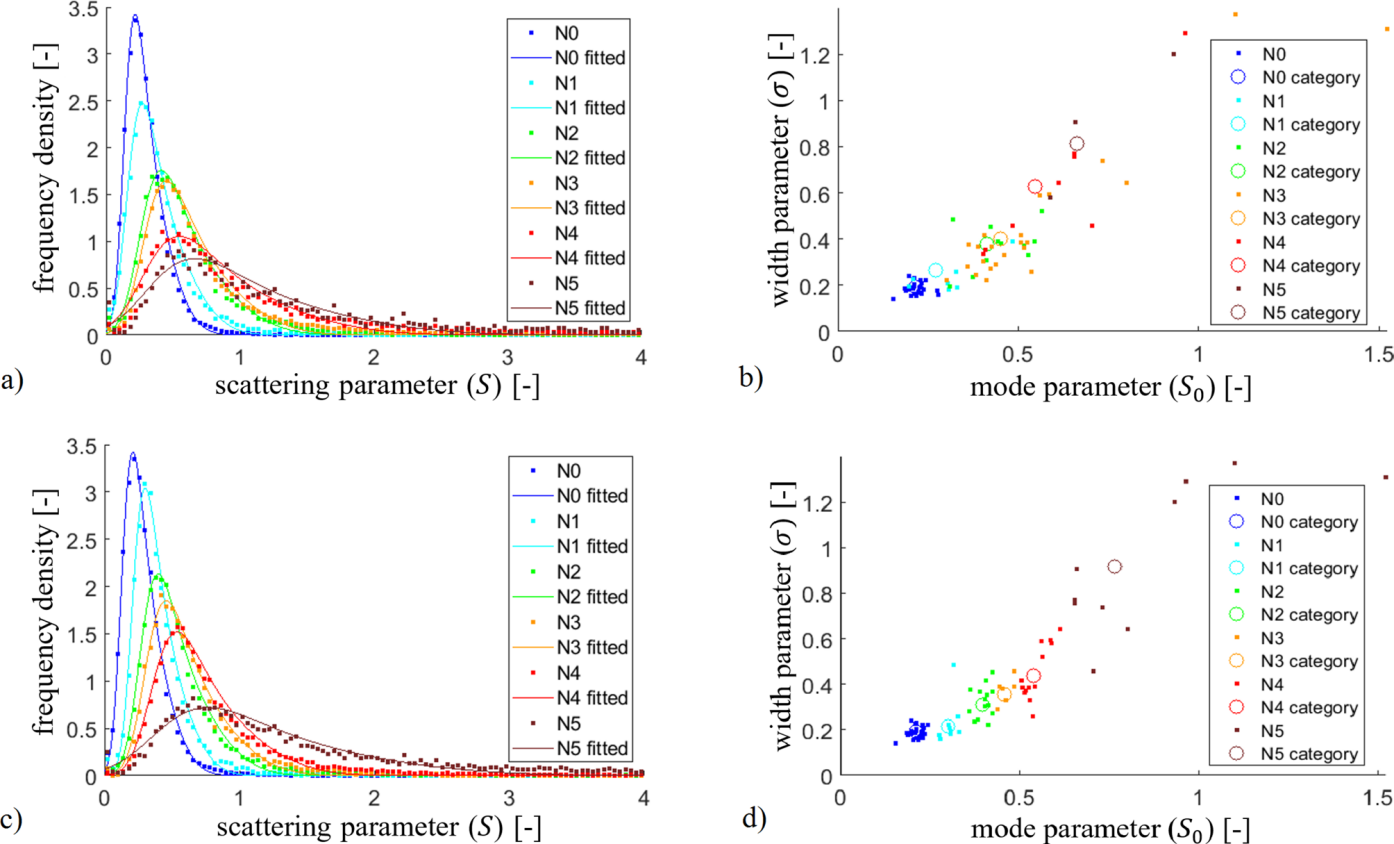
**(a)** Frequency density of microlenses showing the same amount of ocular scattering in cumulative sense (i.e. all measured eyes are included), for different progression of cataract. **(b)** Map of Eq. (4) parameters for each individual eye. The group-specific $S_0$ and $\sigma$ values are indicated for all LOCS III categories as hollow circles. In case of **(a)** and **(b)** LOCS III categories have been assigned to eyes by clinical diagnosis. Diagrams **(c)** and **(d)** show frequency densities and the parameter map after reclassification (by algorithmic category assignment) of the measured eyes, see discussion in the next subsection.

Our clinical tests were performed in a darkened examination room to prevent the infrared spectral components of sunlight from perturbing the sensitive measurement. The exposure duration of the wavefront sensor was set to 120 ms as a trade-off between rapid image capture and sufficient signal-to-noise ratio. In order to enrich the statistics of *S*, at each test multiple images were taken of the same eye. The reason behind this was that saccadic movements always cause a little difference between subsequent images, producing a smoother distribution of *S* (see e.g. Fig.  6a, c). We determined the number of necessary images by evaluating the goodness of the fit of Eq. (4) to the *S* histograms. Figure 5 shows the 95% confidence interval radius of $S_0$ and $\sigma$ as a function of image number acquired from the same eye. According to this analysis we took 10 images of a given eye during each test.

### Raw histograms of the scattering parameter

From the pupil maps we generated frequency density histograms of *S* by considering all data coming from eyes in the same LOCS III N group as a whole (the groups were created based on the slit-lamp reference measurements). These so-called *cumulative histograms* can be seen in Fig. 6a. Formula (4) was fitted to the distribution of each group to determine their group-specific $S_0$ and $\sigma$ quantities. For one individual eye the distribution and the fitted curve are very similar to those of the corresponding group. In order to avoid confusion we decided not to present both, only the cumulative results of the groups.

As a next step, we performed the histogram fit for each measured eye separately, and determined their unique $S_0$ and $\sigma$ values. The result is depicted in the so-called *parameter map*, see Fig. 6b, where each dot corresponds to one eye, and the circles represent the group-specific $S_0$ - $\sigma$ parameter pairs.

The overlapping of the groups shows well that the results of our measurements are definitely mixed up between the neighboring LOCS III N categories. Distributions in Fig. 6a also supports that the categories are not resolved adequately. Since they are based on visual inspection, standard clinical slit-lamp tests are strongly subjective: according to ophthalmologists’ experience, mistakes of ±1 category may usually occur. Consequently, we suppose that our slit-lamp reference measurements represented the major contribution to this error. Not having the possibility to increase the precision of these, we had to find another way by which the application of our method could be demonstrated and its accuracy could be estimated.

### Reclassification

**Figure 7:**
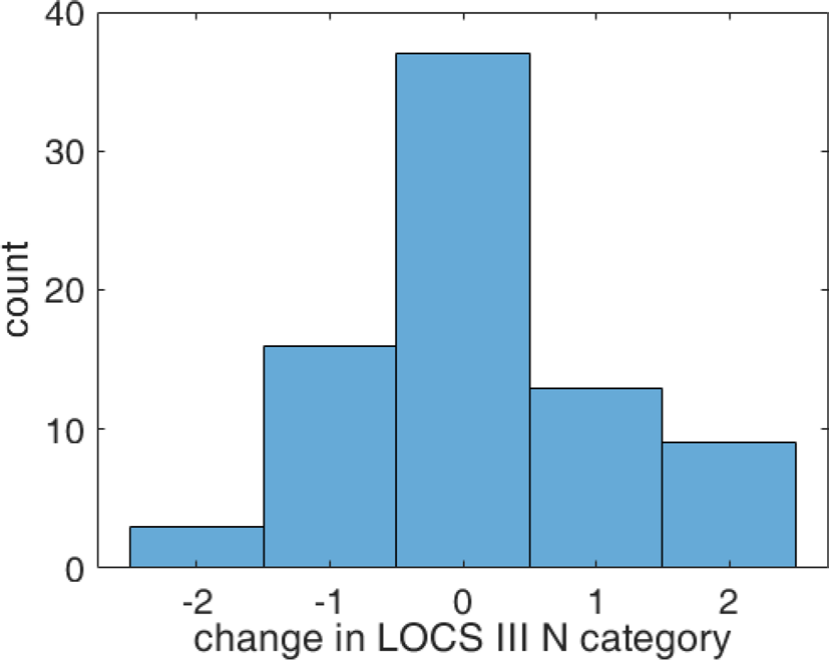
Histogram of reclassified eyes as a function of category displacement for all the examined 78 eyes, from which 53% were reclassified.

If we assume that the $S_0$ parameter quantitatively characterizes the cataract of an eye and that there is a strictly monotonic dependence between the two, we can artificially reduce the uncertainty of reference measurements by a reclassification of slit-lamp LOCS III categories. For this purpose we used the following sequence. First, we formed new groups from those eyes that have a minimum difference between their $S_0$ parameter and a given group-specific $S_0$ value. Second, we re-fitted the new cumulative group histograms to determine the new group-specific $S_0$ parameters. We iterated these two steps until the number of reclassified eyes became zero. The “steady-state” cumulative histograms can be seen in Fig. 6c, where the fitted curves now clearly separate from each other. The amount of reclassified eyes versus category displacement is depicted in Fig. 7, implying that there is only 47% coincidence between the original slit-lamp measurements and the results of our reclassification process.

If we take a look at the new parameter map in Fig. 6d, we see that the reclassified LOCS III groups are now separated (the position of the dots did not change, only their color, i.e. the category number has been updated). This is an inherent property of the described reclassification method: since any point in a category should be placed the closest to its representative group $S_0$ parameter, it cannot happen that another point being even closer to it would be categorized into the neighboring group. The diagram suggests that by characterizing eyes with the mode parameter $S_0$, a definite cataract categorization is possible—at least in theory. We know that the correctness of the above reclassification process is at least questionable according to the standards of measurement technology, thus our results should be strictly regarded as preliminary. Accepting the reclassified LOCS III values as a hypothetical reference, in the next subsection we provide a method to convert the $S_0$ parameter of any eye into LOCS III category.

### Calibration

**Figure 8:**
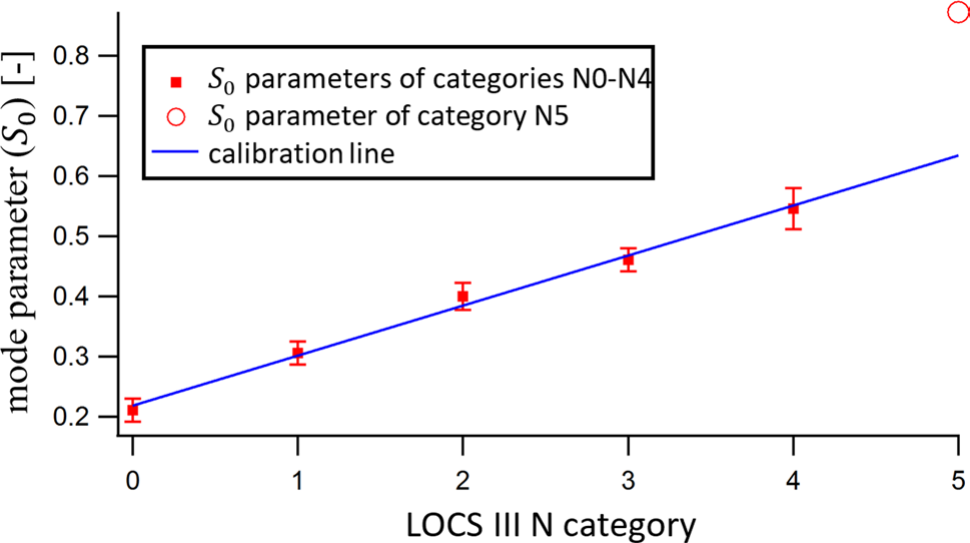
Calibration line fitted to the LOCS III N category—average $S_0$ parameter pairs. The group of N5 category was excluded from the calibration process (see details in the text).

The clinical relevance of our wavefront sensor-based cataract measurement requires the new metric $S_0$ to be related with the widely-used standard LOCS III scale. Therefore, we performed a calibration to seek for an analytic function that best fits to the reference LOCS III category—$S_0$ points. Figure 8 shows the average (dot) and standard deviation (error bar) of $S_0$ parameters in each reclassified group. Justifying our initial assumption about monotony, the trend is apparently linear, the only exception is group N5. We should know about patients with such a high progression of cataract that the intensive scattering in their eye makes their visual characterization rather cumbersome. Hence, during our slit-lamp examinations all those eyes were categorized as N5, the cataract of which *exceeded* the lower limit of scattering corresponding to N5. Since the average $S_0$ of this group is inherently too high, we omitted it from the linear regression when computing the coefficients of the fitting formula. Equation (5) shows the final result, with the 95% confidence intervals indicated. The constant term implies that a small light scattering is always present even in healthy eyes. In case there was a need for more precise figures for the regression formula coefficients, the whole calibration process should be repeated with reference values of lower uncertainty.

5 $$\begin{aligned} S_0=\left( 0.0832\pm 0.0074\right) \cdot LOCS\ III\ N\ category+\left( 0.218\pm 0.016\right) \end{aligned}$$

**Figure 9:**
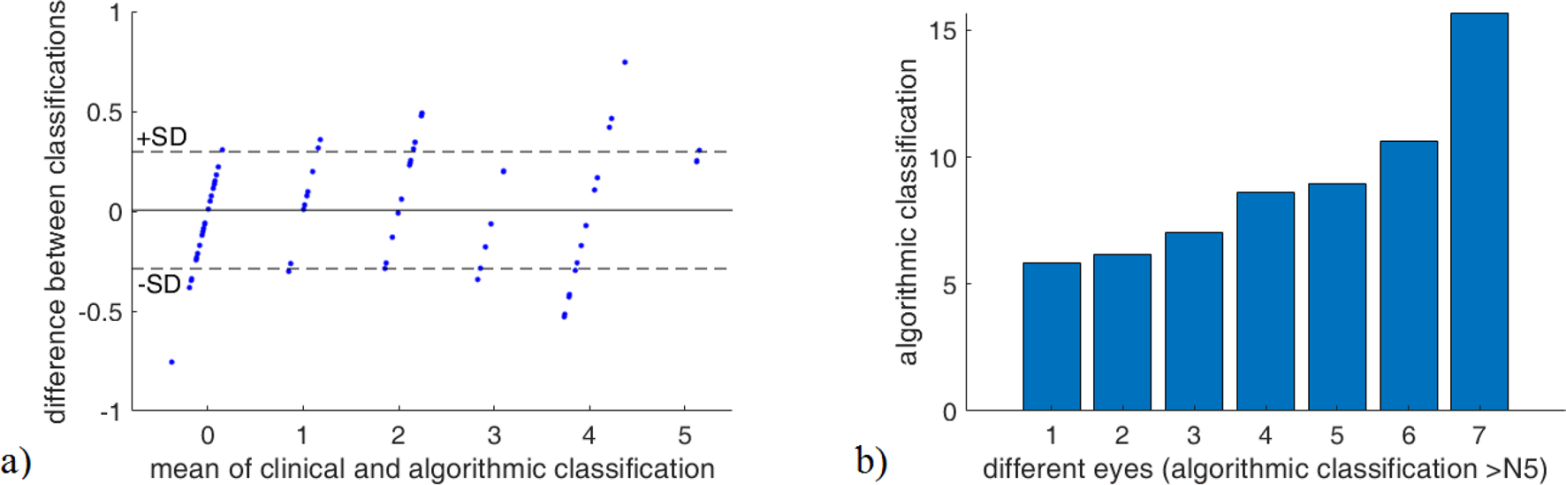
**(a)** Bland–Altmann plot visualising the correspondence between clinical diagnosis and algorithmic classification. Each point shows the LOCS III N category difference for one eye. **(b)** Algorithmic classification of seven eyes clinically diagnosed as N5.

By using the inverted regression formula we calculated the “algorithmic” LOCS III category from the corresponding $S_0$ value far all the 78 examined eyes and compared them to the reclassified clinical reference measurements. Fig. 9a plots the Bland–Altmann analysis that illustrates the similarity between clinical and algorithmic grading. The chopped nature of the diagram comes from the fact that our measurement provides a real decimal number over the LOCS III scale, whereas clinical grading is discrete, i.e. can have only positive integer values. The solid line corresponds to the mean of differences, while dashed lines show the standard deviation of differences: $SD=\pm 0.29$ category. This is the residual error of our calibration, which is very close to $\pm 0.33$, the error reported in extremely accurate slit-lamp measurements used to define the LOCS III standard^4^. This result is very promising, and clearly demonstrates the practical usability of our method. The $SD=\pm 0.29$ value corresponds to the standard deviation of uniform distribution, showing that eyes categorized by our method are evenly distributed inside a group. This is exactly what we expect, if we suppose that randomly selected cataractous patients fill up each group evenly. Since by $\pm 0.29$ we reached the theoretical lower limit of *SD*, a better estimation on accuracy could only be achieved if the reference measurements were made on an extended LOCS III scale containing sub-division between the integer category numbers.

In Fig. 9a, as well as in the above calculations we considered only those eyes that have an algorithmic LOCS III value below 5.5, for the same reasons as at determining the calibrated fitting function. Cataract values of eyes above 5.5 are plotted separately in Fig. 9b, showing that among them there were patients with extremely progressed cataract indeed, as we suspected.

## Conclusions

We developed an efficient method to characterize cataract of human patients objectively and quantitatively based on Shack-Hartmann wavefront sensors. We derived a new quantity called scattering parameter (*S*) by which local scattering of the crystalline lens can be determined along a lateral map of the pupil. The method has no depth-resolution. We suggested the most frequent scattering parameter ($S_0$) in a pupil as a measure to characterize the given eye as a whole. We demonstrated the operation of our method by using a custom-built measuring equipment on a subject pool containing healthy and cataractous eyes of N-type (78 in total). We performed a preliminary calibration so that the system reports the grade of cataract over the LOCS III scale as a real decimal number. We found the connection between the $S_0$ and LOCS III values to be linear. The residual error of the calibration was $SD=\pm 0.29$, less than the error of the most precise clinical measurements^4^. This confirms that our method works, and can be used for the automatic classification of nuclear cataract. Although it requires further studies to reveal the real accuracy, our preliminary results imply that the presented new method promises to be sensitive enough to detect early stages of cataract, and in the future, may be suitable to precisely track the progression of the disease.
